# A Psychological Distress-Linked Plasma Metabolite Score Predicts Cognitive Aging

**DOI:** 10.1093/geroni/igaf122.3691

**Published:** 2025-12-31

**Authors:** Yiwen Zhu, Tianyi Huang, Raji Balasubramanian, Clary Clish, Timothy Hughes, Jerome Rotter, Susan Hankinson, Laura Kubzansky

**Affiliations:** Harvard T.H. Chan School of Public Health, Boston, Massachusetts, United States; National Institute on Aging, Baltimore, Maryland, United States; University of Massachusetts Amherst, Amherst, Massachusetts, United States; Broad Institute of MIT and Harvard, Cambridge, Massachusetts, United States; Wake Forest University School of Medicine, Winston-Salem, North Carolina, United States; The Lundquist Institute at Harbor-UCLA Medical Center, Torrance, California, United States; University of Massachusetts Amherst, Amherst, Massachusetts, United States; Harvard T.H. Chan School of Public Health, Boston, Massachusetts, United States

## Abstract

Chronic psychological distress, such as depression and anxiety, is associated with cognitive decline and increased risk of Alzheimer’s disease and related dementias. We previously developed and validated a plasma metabolite-based distress score (MDS), which predicted incident risks of cardiometabolic conditions in independent samples. Components of the MDS reflect metabolic consequences of chronic distress, such as pathways of inflammation and neurotoxicity. In this study, we examined whether baseline MDS was linked to faster cognitive decline and elevated risk of mild cognitive impairment (MCI) or dementia among 3103 participants (mean age 58.2 years at baseline) from the Multi-Ethnic Atherosclerosis Study (MESA) over 24 years of follow-up. Global cognitive function was assessed using a summary score of the Cognitive Abilities Screening Instrument (CASI) at four time points spanning 10-24 years after baseline. Adjudicated cognitive status was obtained 20 years post-baseline based on expert reviews of clinical and informant materials beyond the CASI. Analyses adjusted for ages at baseline and follow-up, study visit, test language, gender, race/ethnicity, socioeconomic status, and a range of biobehavioral and health-related factors. High MDS at baseline was associated with faster declines in global cognitive function measured by repeated CASI assessments (beta coefficient from a linear mixed model for the interaction between MDS and age=-0.03, 95% CI[-0.05, -0.004], p = 0.02) and slightly higher odds of MCI or dementia (logistic regression Odds Ratio=1.13[1.00, 1.27], p = 0.05). Metabolic alterations linked to psychological distress may contribute to accelerated cognitive aging. Characterizing these pathways could inform strategies to mitigate the impact of distress on cognitive health.

